# Characteristics of Patients With Community‐Acquired Pressure Injuries

**DOI:** 10.1002/nop2.70128

**Published:** 2024-12-30

**Authors:** Midori Nagano, Yoshiko Kubo, Akiko Egawa, Masayo Kobayashi, Masami Sato

**Affiliations:** ^1^ The Jikei University School of Nursing Tokyo Japan; ^2^ Faculty of Nursing Kyouritu Women's University Tokyo Japan; ^3^ Nursing Department The Jikei University Hospital Tokyo Japan; ^4^ Nursing Department The Jikei University Daisan Hospital Tokyo Japan

## Abstract

**Aim:**

(1) To classify patients with community‐acquired pressure injury (CAPI) according to the risk factors of PI and to assess validity of the classified groups. (2) To clarify characteristics of each group for CAPI prevention and care.

**Design:**

This study is designed to classify CAPI patients into clusters based on a retrospective study of medical records, followed by cluster analysis and description of each cluster's characteristics.

**Methods:**

Risk factors and status of CAPI, as well as discharge destination, were investigated based on 1 year's worth of medical records of patients with CAPI on hospital admission during 2018–2019. After calculating descriptive statistics, cluster analysis was conducted by Ward's method of Euclidean distance referring to risk factors of PI. Lastly, each of the defined clusters underwent multiple comparisons.

**Results:**

From 324 patients with CAPI, 272 patients were selected as the study subjects, due to availability of sufficient information regarding risk factors of PI. After classification into three groups, data were interpreted by Euclidean distance and comparison between ‘attribute and risk factors of PI’ and ‘PI and destinations after discharge’.

**Patient or Public Contribution:**

Patients with CAPI were classified into three clusters and validity of the classification was assessed. Patients who had ‘maintained ADL’, as well as cognitive status, were expected to be capable of self‐care and self‐management. Patients with ‘low ADL’ were characterised by insufficient self‐care or home care resulting in having CAPI and would require aged care service. Patients who were at ‘very high risk’ of having PI were characterised by incurring significant burden on caregivers and need of medical services that prospects terminal care.

## 1 Introduction

The ratio of aged people in the world population is expected to continue growing until 2100 (United Nations Population Division n.d.). Ageing can not only cause changes in skin conditions but also comorbidities and impaired mobility, and thus, increase the risk of acquiring a pressure injury (PI), being the key indicator of quality of care (Hahnel et al. 2017; Cooper et al. 2015; Power, Stewart, and Brotherton 2012). It is said that PI causes negative impacts on patients' financial conditions and on outcomes of PI care (Demarré et al. 2015; Nguyen, Chaboyer, and Whitty 2015; Latimer, Chaboyer, and Gillespie 2014). In spite of many studies about PI preventive care in acute‐care hospitals, there are few studies on PIs at community settings, that is, places where patients stayed before hospital admissions such as home and aged care facilities. It has been pointed out that conditions of hospital‐acquired pressure injuries (HAPIs) are different from community‐acquired pressure injuries (CAPIs). Therefore, study on patients with CAPI can lead to the development of new methodologies for CAPI prevention and treatment (Singh and Shoqirat 2021).

## 2 Background

In 2002, system to prevent and treat PIs became one of the standards for hospitals and aged care facilities in Japan (Ministry of Health, Labour and Welfare of Japan 2002). In 2005, the Japanese Society of Pressure Ulcers (JSPU) proposed the ‘Guideline for Prevention and Management of Pressure Ulcers’, enhancing measures against PIs at hospitals and facilities (Japanese Society of Pressure Ulcers Guideline Revision Committee 2016). According to a survey conducted in 1997, PIs occurred in 2.8% of the patients admitted to hospitals, while a survey in 2021 found that cases of PIs decreased to 2.03% (Ishikawa and Miyachi 1996; Fact‐finding Committee of the Japanese Society of Pressure Ulcers 2023). Incidence of PIs at general hospitals in Japan was assumed to have decreased from 1.18% to 1.15%, whereas prevalence of diseases increased from 2.24% to 2.37% during 2013–2021. In the same period, the ratio of patients with a CAPI increased from 19.8% to 49.8%, indicating that an increase of PI occurrence in general hospitals was due to increases in PIs occurring outside hospitals.

CAPI has also been reported to have high prevalence in acute‐care hospitals around the world. In Australia, the number of patients with CAPI admitted to acute‐care hospitals accounted for 6.6% of those who were aged 65 or over with impaired mobility (Latimer et al. 2019a). Among patients admitted to surgical ward and critical care unit in acute‐care hospitals in the US, 3.3% were reported to have CAPI (Alderden et al. 2020). In acute‐care hosipitals in Italy, 14.9% of patients in the medical ward had CAPI (Sanson et al. 2021). According to a study in China, 4.3% of inpatients aged 65 or over were found to have CAPI (Zhang, Yang, and Luo 2021). In Singapore, 46% of admitted patients with PI were reported to have CAPI, similarly to Japan (Aloweni et al. 2024). Even though these studies could not be directly compared owing to different investigation criteria, these numbers suggest the necessity of measures against CAPIs.

Risk factors of CAPI were reported to be main characteristics seen in older people, including living home alone and residing at aged care facilities (Worsley et al. 2016; Corbett et al. 2017). In addition, having more than one complication and receiving palliative care were also raised as risk factors of CAPI (Hahnel et al. 2017; Jaul and Calderon‐Margalit 2015; Jaul, Meiron, and Menczel 2016). In China, risk of having CAPI was found to be higher in males (Zhang, Yang, and Luo 2021). Unlike the normal PIs, patients with CAPI were said to have dry skin and higher risk of having HAPI, as well as incurring higher medical costs (Sanson et al. 2021; Reilly et al. 2021). Since CAPI is a PI which occurs at home or at a residential facility such as aged care facilities, nurses who work in hospitals are unable to take preventive or treatment measures. Among all nurses in Japan, only around 8% are community nurses, making it difficult to manage CAPI for all patients at community settings (Japanese Nursing Association n.d.). While approximately 80% of nurses in Japan are mainly working in hospitals, we believed that these nurses can also help with care and prevention of PIs occurring outside hospitals. Hence, we classified and identified patients whose CAPIs were preventable at hospitals. Classifying and establishing measures against CAPI can be of significant benefit both in Japan and in other countries where the ageing population is growing.

## 3 The Study

### 3.1 Aim and Objective

This study aimed to classify patients with CAPI and to examine treatment and preventive measures in accordance with characteristics of their conditions. From this study, there were two specific outcomes to be achieved: (1) Classification of CAPI patients based on risk factors of PI acquired before and during hospitalisation, followed by validation of the classified conditions of PIs on admission and discharge, as well as destination after discharge; (2) Clarification of characteristics that explain prevention and treatment measures of CAPI for each of the classified groups.

### 3.2 Technical Terminology Used to Describe the Aim

The subject patients for this study had PIs not only at home but also at aged care facilities and other healthcare institutions. Hence, patients with CAPI were operationally defined as ‘patients with PIs at the time of hospital admission disregarding the location of acquisition’.

## 4 Methods

In this study, medical records of patients with CAPI were used for retrospective cross‐sectional study, while complying with Strengthening the Reporting of Observational Studies in Epidemiology (STROBE) guideline (Checklist of cross‐sectional studies. See Supporting Information 1). The study subjects were classified into three clusters and compared as a part of analysis to understand the characteristics of patients with CAPI.

Study subjects were identified from 1 year's worth of medical records of patients with CAPI admitted to four hospitals: an advanced treatment hospital located in central part of metropolitan area (A), an emergency and critical care centre (B) and two community‐based hospitals (C and D) in suburban areas (total number of beds: 2689). The study periods were from May 2018 to April 2019 in Hospitals A, B and D and from November 2018 to October 2019 in Hospital C. The sample size was determined based on the number of corresponding cases within the study periods. For patients with PIs who were hospitalised more than once during the study period, only the record of latest admission was used. The medical record used in this study was a comprehensive electronic medical record, including all clinical records and examination data provided by medical specialists, such as doctors, nurses, dietitians, physiotherapists and pharmacists. Moreover, to determine the ratio of patients with CAPIs admitted to hospitals, all registered beds and newly hospitalised patients during the study period were also investigated.

Survey items are set as shown in Table 1 upon consensus of an expert panel of eight wound, ostomy and continence nurses (WOCNs) and three clinical nursing researchers from four acute‐care hospitals. Information for the study items were the status of PIs and risk factors found in individual patients, obtained from the medical records. Risk factors of PI were also examined based on the findings from preceding studies, including Braden Scale, OH Scale, K‐Scale, Risk Factor Assessment Form for Pressure Ulcers, Mini Nutritional Assessment (MNA), Subjective Global Assessment (SGA) and daily life independence level (Guigoz and Vellas 1999; Banks et al. 2010; Takigawa 1994). To investigate ‘PIs on hospital discharge’, comparisons were made between data of PIs assessed just prior to discharge and DESIGN‐R data collected when PIs were at their worst condition, and were rated as either ‘improved’, ‘worsened’ or ‘unknown’ by WOCN, referring to the ‘DESIGN tool’ (Japanese Society of Pressure Ulcers 2014).

**TABLE 1:** Attribute and risk factors of CAPI.

Study items	*n*	(%)	*N*	
	Median	Range		
Attribute and risk factors				
Attribute				
Gender (male)	135	(49.6)	272	
Age ≧ 79	139	(51.1)	272	○
Age	79	1–117	272	
Before admission				
Residency				
Home	211	(77.9)	272	○
Aged care facility	32	(11.8)	272	
Hospital	29	(10.7)	272	
Within 1 month				
Outpatient consultation	135	(49.6)	272	○
Chemotherapy	20	(7.4)	272	○
Within 1 week				
Daily life independence level ≧ C^a^	97	(35.7)	272	○
Sudden change of ADL	119	(43.8)	272	○
On admission				
Conditions				
Emergency	226	(84.0)	269	○
Pneumonia	72	(26.5)	272	○
Urinary tract infection	35	(12.9)	272	○
Dementia	80	29.4)	272	○
PI risk assessment				
Daily life independence level ≧ C^a^	183	(67.3)	272	○
Poor mobility	168	(61.8)	272	○
Morbid bony prominence	121	(44.5)	272	○
Joint contracture	64	(23.5)	272	○
Malnutrition	187	(68.8)	272	
Skin moisture (hyperhidrosis and incontinence)	174	(64.0)	272	○
Oedema	102	(37.5)	272	○
History of skin tear	107	(39.3)	272	○
Nutritional assessment				
BMI	19	9–35	218	
SGA value^b^	7	1–14	132	
SGA rating (severe malnutrition)	106	(39.0)	272	○
PI and destination after discharge				
PI				
On admission				
Number	1	1–6	266	
Area (sacral region)	109	(40.1)	272	
Depth ≧ Grade III	81	(18.2)	272	
Total score of DESIGN‐R	7	0–49	264	
On discharge				
PI	114	(41.9)	272	
Length of hospital stay	26	1–290	272	
Place of residence after discharge				
Home	111	(40.8)	272	
Aged care facility	56	(20.6)	272	
Hospital	48	(17.6)	272	
In‐hospital mortality	54	(19.9)	272	

Abbreviation: ○, items to include in hierarchical clustering.

^a^ C: Bedridden and requires full assistance.

^b^ Two hospitals did not calculate.

The WOCNs of PI team in the subject hospitals collected the data used for setting the survey items by extracting data from the list of patients with PIs shared in the team.

In the subject hospitals, PIs were measured exclusively by WOCNs. They collected the data used for setting the survey items to investigate by extracting data of patients with CAPIs from the list of patients with PIs shared in the team.

The subject patients for this study had PIs not only at home but also at aged care facilities and other healthcare institutions. Hence, patients with CAPI were operationally defined as ‘patients with PIs at the time of hospital admission disregarding the location of acquisition’.

After the calculation of numbers (percentages) as nominal variables and medians (interquartile ranges) as continuous variables, cluster analysis was conducted using Euclidean distance and Ward's method based on risk factors of PI, while considering multicollinearity.

To classify patients based on risk factors of PIs, cluster analysis was conducted by applying the following study items: Age ≧ 79; (residency before admission) home; (Within 1 month prior to admission) outpatient consultation and chemotherapy; (Within 1 week prior to hospital admission) daily life independence level ≧ C and sudden change of ADL; (on admission) emergency, pneumonia, urinary tract infection, dementia, daily life independence level ≧ C, poor mobility, morbid bony prominence, joint contracture, skin moisture, oedema, history of skin tear and SGA rating (marked ‘○’ in Table 1). Referring to the extracted dendrogram, number of clusters were set, followed by multiple comparisons between ‘attribute and risk factors of PI’ and ‘PI and destination after discharge’ in order to clarify the characteristics of patients with CAPIs in each cluster. Multiple comparisons were performed using a Kruskal–Wallis test as normal distribution was not seen in all study items such as risk factors of PI, progress of PI and destination after discharge. To determine statistical significance, alpha level of two‐sided test was set as *p* < 0.05. All analyses were implemented using IBM SPSS Statistics 27.

Investigation started after approval by Research Ethics Committee in institutions (approval number: 35‐442), where members of this study and heads of the institutions were present. Overview of the study was released on the homepage of each institution. A correspondence table of the subjects of this study was made anonymously, and the data were extracted using a computer disconnected from external networks. The extracted data were stored under strict control.

## 5 Results

The study period at Hospitals A, B and D were from May 2018 to April 2019, while Hospital C was from November 2018 to October 2019. Collectively, there were 64,898 patients in total who were newly admitted during the study period, where the total number of patients with CAPI was 324. However, eight patients were admitted twice and three patients were admitted three times during the study period. The final number of patients with CAPI identified was 311, and 272 patients with sufficient information regarding risk factors of PI were further selected as study subjects.

The age of the subjects ranged from 1 to 117 with median age of 79 years old. Of PIs found on admission, 81.8% were in Grade II or lower, indicating that damage was limited to epidermis or dermis. Patients who were admitted from home accounted for 77.9%, while 11.8% were from aged care facilities, and 10.7% were from other hospitals. At the time the patients admitted to hospitals, 35.7% of them were bedridden and required the full extent of aged care; 29.4% had symptoms of dementia; 64% had risk of having skin moisture from wearing adult diapers; and 39% were severely malnourished, indicating that the patients are at risk of having PIs as shown in Table 1.

### 5.1 Subjects (Cluster Analysis of Patients With CAPI)

**FIGURE 1:**
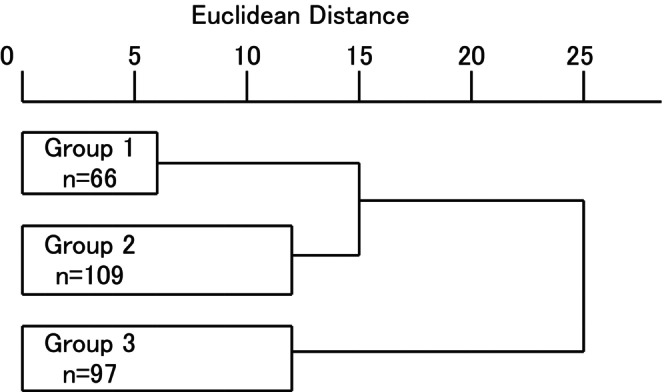
Dendrogram of patients with CAPI by clustering.

Cluster analysis of 272 patients classified by risk factors of PI immediately before and after admitting to hospital resulted in classification into three groups: 66 patients in Group 1, 109 patients in Group 2 and 96 patients in Group 3 (Figure 1). As shown in Table 2, patients in Group 3 had the highest risk of PI, followed by Group 2 and Group 1. In Group 1, 98.5% lived at home, 81.8% had outpatient consultations within a month before admission, and 27.3% were undergoing chemotherapy, but only 10.6% were scored C in daily life independence level. Furthermore, 98.5% had no symptoms of dementia, with very few patients needing aged care facilities. In Group 2, 42.2% of the patients were scored C in daily life independence level due to limited activities of daily living (ADL). Additionally, 25.7% of Group 2 patients had symptoms of dementia, 78.9% resided at home, and 42.2% had outpatient consultations within a month before admission and none were receiving chemotherapy. In Group 3, 62.9% were admitted from home and 28.6% were admitted from an aged care facility. The median age of Group 3 was 85 years old, where 52.6% had symptoms of dementia. In addition, risk of having skin moisture, mainly from wearing adult diapers, was found in 88.7% of Group 3 patients, and 60.8% were found to have severe malnutrition by SGA.

**TABLE 2:** Attribute and risk factors of PI in each group classified by clustering.

	Group 1		*N*	Group 2		*N*	Group 3		*N*	Group comparison
	Median	Range		Median	Range		Median	Range		*p* *
	*n*	(%)		*n*	(%)		*n*	(%)		
Attribute										
Gender (male)	34	(51.5)	66	55	(50.5)	109	46	(47.4)	97	0.856
Age ≧ 79	18	(27.3)	66	48	(44.0)	109	73	(75.3)	97	< 0.001
Age	74	41–92	66	76	1–98	109	85	47–117	97	< 0.001
Before admission										
Place of residence										
Home	65	(98.5)	66	86	(78.9)	109	61	(62.9)	97	< 0.001
Aged care facility	0	(0.0)	66	4	(3.7)	109	28	(28.9)	97	< 0.001
Hospital	1	(1.5)	66	19	(17.4)	109	8	(10.3)	97	< 0.001
Within 1 month										
Outpatient consultation	54	(81.8)	66	46	(42.2)	109	35	(36.1)	97	< 0.001
Chemotherapy	18	(27.3)	66	0	(0.0)	109	2	(2.1)	97	< 0.001
Within 1 week										
Daily life independence level ≧ C^a^	7	(10.6)	66	46	(42.2)	109	44	(45.4)	97	< 0.001
Sudden change of ADL	28	(42.4)	66	44	(40.4)	109	47	(48.5)	97	0.491
On admission										
Conditions										
Emergency	46	(69.7)	66	89	(83.2)	107	91	(94.8)	96	< 0.001
Pneumonia	14	(21.2)	66	21	(19.3)	109	37	(38.1)	97	0.005
Urinary tract infection	2	(3.0)	66	12	(11.0)	109	21	(21.6)	97	0.002
Dementia	1	(1.5)	66	28	(25.7)	109	51	(52.6)	97	< 0.001
PI risk assessment										
Daily life independence level ≧ C^a^	23	(34.8)	66	79	(72.5)	109	81	(83.5)	97	< 0.001
Poor mobility	11	(16.7)	66	71	(65.1)	109	86	(88.7)	97	< 0.001
Morbid bony prominence	40	(60.6)	66	31	(28.4)	109	50	(51.5)	97	< 0.001
Joint contracture	9	(13.6)	66	24	(22.0)	109	31	(32.0)	97	0.023
Malnutrition	45	(68.2)	66	47	(43.1)	109	95	(97.9)	97	< 0.001
Skin moisture	27	(40.9)	66	61	(56.0)	109	86	(88.7)	97	< 0.001
Oedema	32	(48.5)	66	14	(12.8)	109	56	(57.7)	97	< 0.001
History of skin tear	15	(22.7)	66	24	(22.0)	109	68	(70.1)	97	< 0.001
Nutritional assessment										
BMI	18	11–33	61	20	9–33	89	20	14–35	68	0.157
SGA value	10	2–14	35	8	1–14	48	5	1–11	49	< 0.001
SGA rating (severe malnutrition)	18	(27.3)	66	29	(26.6)	109	59	(60.8)	97	< 0.001

^a^ C: Bedridden and requires full assistance.

* Kruskal–Wallis test.

The score of DESIGN‐R was the highest in Group 3, indicating the most severe conditions of PI, followed by Group 2 and Group 1 (Table 3). The median DESIGN‐R score of PI was 4 in Group 1, demonstrating low severity, and home was the place where patients more likely reside after discharge, which accounted for 57.7%. However, 22.7% of the patients had poor general condition and died in hospital. The median DESIGN‐R score in Group 2 was 8, indicating a similar high severity to Group 3. Depth of PIs in Grade III or higher were found in 28.4% of the patients in Group 2, which was less than Group 3 (39.2%). While not stated in Table 3, we found that Group 2 included four patients admitted from aged care facilities and one died in hospital during hospitalisation, while 28 patients went to aged care facilities after discharge. Of the 28 patients who resided in aged care facilities after discharge, 25 patients (22.9%) took hospitalisation as an opportunity to consider living outside their homes. At the time of discharge, 50.2% of patients in Group 3 still had PIs, 23.7% returned home after discharge and 28.9% died in hospital.

**TABLE 3:** PI and destination after discharge in each group classified by clustering.

	Group 1		*N*	Group 2		*N*	Group 3		*N*	Group comparison
	Median	Range		Median	Range		Median	Range		*p* *
	*n*	(%)		*n*	(%)		*n*	(%)		
PI										
On admission										
Number	1	1–3	63	1	1–6	106	1	1–6	94	0.264
Area (sacral region)	29	(43.9)	66	36	(33.0)	109	44	(45.4)	97	0.151
Depth ≧ Grade III	12	(18.2)	66	31	(28.4)	109	38	(39.2)	97	0.015
Total score of DRSIGN‐R	4	0–31	63	8	0–9	104	8	3–45	97	0.008
On discharge										
PI	23	(34.8)	66	42	(38.5)	109	49	(50.5)	97	0.091
Length of hospital stay										
	22	2–164	66	29	3–290	109	21	1–172	97	0.129
Place of residence after discharge										
Home	38	(57.6)	66	50	(45.9)	109	23	(23.7)	97	< 0.001
Aged care facility	5	(7.6)	66	28	(25.7)	109	23	(23.7)	97	0.010
Hospital	8	(12.1)	66	19	(17.4)	109	21	(21.6)	97	0.294
In‐hospital mortality	15	(22.7)	66	11	(10.1)	109	28	(28.9)	97	0.003

* Kruskal–Wallis test.

## 6 Discussion

In order to clarify characteristics of patients with CAPI, 272 subject patients were classified into three groups by cluster analysis: ‘Group 1: Maintained ADL’, ‘Group 2: Low ADL’ and ‘Group 3: Very high risk’. This section presents the validity of the said cluster classifications, as well as characteristics of PI and measures taken in each group.

1. Validity of classification
  Validity of the three clusters were assessed by examining the results of cluster analysis and data interpretation.
  According to the dendrogram of patients with CAPI (Table 1), patients were classified into two major groups, where the first cluster was the combination of Group 1 and Group 2, and the other cluster was Group 3. Risk factors of PI such as older age and nutritional status were predominant in Group 3 compared to Groups 1 and 2 (Table 2). Therefore, patients in Group 3 were at very high risk of having PIs. Group 3 also had difficulty in self‐care because of impaired mobility and cognitive dysfunction that incurs a significant burden of caregiving and required specialised pressure‐relieving devices. For the provision of full‐scale aged care services and pressure relief, identification and classification of patients at high risk, like those in Group 3, was believed to be meaningful. Although patients in Group 1 and Group 2 had similar age and nutritional status, there was a significant difference in ADL and symptoms of dementia, which critically impact health‐related functions including self‐care and self‐management. Since methods of supporting daily lives largely differ between Group 1 and Group 2, classification of a cluster was meaningful. According to the analysis based on Euclidean distance and risk factors of PI, as well as data implementation related to the patients with PI and their daily lives, the three clusters were considered to be valid.
  Data interpretation of clusters was conducted by comparing ‘PI and destination after discharge’, which were not included in clustering. As shown in Table 3, many of PIs found in Group 3 were severe and deep. More than half of the patients in Group 3 did not have their PIs heal at the time of hospital discharge, causing high medical costs and overload for caregivers after discharge. In Group 2, a high ratio (22.9%) of patients lived at home before admission but moved to aged care facilities after discharge. When capability of self‐care by patients themselves or aged care by family members at home was assessed by nurses and medical social workers (MSW) at hospital discharge, it was suggested that the patients were at the threshold of whether aged care facility is required or not. Many of the patients in Group 1 had a PI at the time of hospital discharge or died during hospitalisation and with some also receiving chemotherapy and outpatient consultation after discharge. Hence, Group 1 was characterised by having severe state of PIs associated with cancer, chronic disease or treatment for such diseases. Furthermore, Group 1 also included patients with diabetes, cardiovascular disease and cerebrovascular disease, which are common in older age and known as risk factors of PI (Kurashige, Naito, and Miyasaka 2018).
  Data interpretation can be done not only from ‘attribute and risk factors of PI’, but also from ‘PI and destination after discharge’. Thus, the three clusters were considered as valid.
2. Characteristics of PI and measures taken in each group.
  (1) Patients with CAPI and maintained ADL (Group 1)
    Group 1 had fewer patients with symptoms of dementia, and therefore more returned home after discharge. These patients were thought to be able to manage their disease by seeing their doctors or re‐admitting to hospital repeatedly.
    Only 10% of patients in Group 1 were bedridden or graded C in daily life independence level 1 week prior to admission. However, 42% had a sudden decrease in ADL a few days before admission and became bedridden. Patients in Group 1 who visited the hospital within a month before admission accounted for 81.8%, and 27.3% had chemotherapy within a month. This indicated that majority of Group 1 patients had a chronic disease, including malignant tumours. Even though the mean age of Group 1 was younger than other groups and had shallow depth of PIs, 22.7% died in hospital, indicating the severity of the disease that caused hospitalisation. Among these patients, CAPIs occurred from having vulnerable tissues associated with main disease and its treatment, as well as skeletal structure at high risk of PIs, such as morbid bony prominences (Mizogami 2019).
    Prevention measures of CAPI for patients like in Group 1 can be carried out by preparing care plans tailored for each individual, focusing on self‐management capability, disease, treatment and areas at high risk of having PI. Providing guidance for hospital discharge by working together with patients to relieve or protect from pressure on areas at risk, which differs between individuals, was expected to allow the patients to voluntarily keep themselves from acquiring PIs. Doctors and nurses in outpatient hospitals, nutritionists or pharmacists can also help patients to prevent CAPI after discharge by providing continual guidance, checking the PI prevention measures, encouragement and assessment of skin conditions, given that patients themselves or their family are able to manage healthcare.
  (2) Patients with CAPI and low ADL (Group 2)
    In Group 2, 42.2% of the patients had a low daily life independence level before hospital admission, 22% had joint contracture, and 56% were at risk of skin moisture from the use of adult diapers. This was indicative that many of the patients were bedridden for at least 2 weeks before admission. Group 2 had slightly lower scores in history of pneumonia, skin tears and malnutrition compared to Group 1. In‐hospital mortality in Group 2 (10%) was found to be lower than other groups. Therefore, a risk factor of PI in Group 2 was assumed to be the low ADL, rather than intrinsic factors such as nutritional status, disease and treatment. Moreover, 42.2% attended outpatient consultations within a month prior to admission and 25.7% had symptoms of dementia, suggesting that many of the patients in Group 2 had difficulty with regular hospital visits as a result of ongoing low ADL.
    Twenty‐five patients (22.9%) in Group 2 changed their place of residence to aged care facilities after discharge, and some had CAPI as a consequence of difficulties with self‐care or work overload for family caregivers. Therefore, when designing a life support plan after hospital discharge including measures to prevent CAPI for patients in Group 2, it is crucial to carry out an assessment to determine which healthcare or welfare services and care systems need to be introduced.
  (3) Patients with CAPIs who are at very high risk of PI recurrence (Group 3)
    Compared with Groups 1 and 2, Group 3 had higher median age by around 10 years, and many had dementia, severe malnutrition and a higher rate of in‐hospital mortality (28.9%) (Table 2). Thus, Group 3 was a cluster that included end‐of‐life patients. The numbers of patients who were admitted from a home setting and resided at home after discharge were considerably fewer in Group 3 than in Groups 1 and 2, suggesting that many Group 3 patients were dependent on aged care or healthcare services, imposing significant burden on caregivers. With regard to caregiving for the CAPI patients admitted from aged care facilities, who formed the majority of this cluster, Tate et al. (2023) and Nagano et al. (2022) articulated the necessity of managing body pressure and nutrition, along with measures to protect against infectious diseases. Consequently, preventing patients in Group 3 from acquiring PIs would require a comprehensive level of support, including nutritional management and infection‐preventing care, on top of installation of a highly functional pressure‐relieving mattress or bed, combined with physical repositioning by caregivers. Such patients require more complex strategic care planning especially at the time of discharge. For some end‐of‐life patients who acquired Kennedy terminal ulcers (KTU), known as unpreventable ulcers, palliative care based on patient's requests were more likely prioritised, rather than practising PI treatment (Kennedy 1989; Latimer et al. 2019b).
    Furthermore, these patients were heavily dependent on medical services and required in‐depth consideration, associated with decision‐making from many perspectives including quality of life (QOL), prevention and treatment of PIs. Taken together, Group 3 was a cluster that required extensive life support after hospital discharge. This can be realised by collaboration of medical specialists with PI management and prevention expertise and community‐based comprehensive care teams including specialists of end‐of‐life care and spiritual care.

### 6.1 Strength and Limitations of the Work

The results of this study were limited to specific communities and cultures, as the subject patients were those who had PI at the time of admission only in four hospitals located in Tokyo. Additionally, there was a lack of preceding studies that specifically classify CAPI, thus making it difficult to validate the study. However, an investigation by the United Nations revealed that countries with average life expectancy of over 80 years in both males and females also exist across Asia, Europe and North America (United Nations iLibrary n.d.). Hence, it is likely that such countries have common characteristics among patients with CAPI. According to a global surveillance of trends in cancer survival, Japan scored higher in survival rate of gastric cancer, liver cancer and lung cancer compared with western countries, while survival rate in other cancers were about the same (Allemani et al. 2018). This suggests that situations and treatment measures of patients with CAPI associated with diseases such as cancer may be similar in some regions.

### 6.2 Recommendations for Further Research

CAPIs tend to occur at home, where medical specialists can seldom intervene to support. To prevent or treat CAPI, we need to assess different care strategies according to characteristics of each patient with CAPI. This study presents supporting information for such strategies by classifying patients with CAPI into three clusters. Patients in Group 1 who visited outpatient hospitals regularly had a higher chance of getting direct advice or intervention by medical specialists, even though they had CAPI. For Group 1, CAPI could be prevented or treated by the patients themselves. Patients in Group 2 required a change of environment, as the patients could no longer look after themselves or home care became a significant burden for caregivers. For such patients, providing aged care support is believed to be a great measure in the prevention of CAPI. Group 3, which consists of patients at high risk, needed advanced medical services and facilities that could accommodate various levels of care while respecting dignity of patients and their family. Continuing studies through interventions to each cluster can lead to an improved quality of healthcare practices for elderly and patients with chronic disease, alongside better PI prevention and treatment measures.

## 7 CONCULUSION

Clustering and comparisons based on data obtained by retrospective study on medical records of 272 patients with CAPI identified three clusters with different characteristics: Group 1 (maintained ADL) with 66 patients, Group 2 (low ADL) with 109 patients and Group 3 (very high risk of PI recurrence) with 97 patients. Euclidean distance and comparisons between ‘attribute and risk factors of PI’ and ‘PI and destination after discharge’ confirmed that data implementation was applicable and proved the validity of the three clusters.

Patients in Group 1 were able to maintain ADL with regular outpatient consultation for chronic disease, but suddenly became bedridden and hospitalised due to acute exacerbation, causing shallow depth CAPI. Since these patients did not have cognitive dysfunction, they were expected to be able to care and manage themselves.

Group 2 consisted of patients with low ADL, acquiring CAPI from being bedridden for extended periods of time before hospitalisation and from wearing adult diapers. This group also had dementia and impaired mobility caused by joint contracture. The patients and their family members were no longer capable of self‐care or home care, resulting in the consideration of aged care services.

Group 3 consisted of debilitated elderly patients with low ADL, severe malnutrition and dementia that collectively confer a high risk of acquiring PIs. These patients most likely caused significant burden on caregivers and required medical services that prospects terminal care.

## Author Contributions

M.N. contributed to supervision and writing. Y.K. contributed to formal analysis and review. A.E. contributed to investigation. M.K. contributed to investigation. M.S. contributed to funding acquisition and review.

## Conflicts of Interest

The authors declare no conflicts of interest.

## Supporting information


Supporting Information 1.

## Data Availability

The data that support the findings of this study are available from the corresponding author upon reasonable request.
